# TB-DROP: deep learning-based drug resistance prediction of *Mycobacterium tuberculosis* utilizing whole genome mutations

**DOI:** 10.1186/s12864-024-10066-y

**Published:** 2024-02-12

**Authors:** Yu Wang, Zhonghua Jiang, Pengkuan Liang, Zhuochong Liu, Haoyang Cai, Qun Sun

**Affiliations:** 1 Key Laboratory of Bio-Resources and Eco-Environment of the Ministry of Education, College of Life Sciences, Sichuan University, Chengdu, 610064 China; 2 Zhejiang Yangshengtang Institute of Natural Medication Co., Ltd, Hangzhou, China; 3 Center of Growth, Metabolism and Aging, Key Laboratory of Bio-Resources and Eco-Environment of the Ministry of Education, College of Life Sciences, Sichuan University, Chengdu, 610064 China

**Keywords:** *Mycobacterium tuberculosis*, Deep learning, Drug resistance, Whole genome mutations, Graphical tool

## Abstract

The most widely practiced strategy for constructing the deep learning (DL) prediction model for drug resistance of *Mycobacterium tuberculosis* (MTB) involves the adoption of ready-made and state-of-the-art architectures usually proposed for non-biological problems. However, the ultimate goal is to construct a customized model for predicting the drug resistance of MTB and eventually for the biological phenotypes based on genotypes. Here, we constructed a DL training framework to standardize and modularize each step during the training process using the latest tensorflow 2 API. A systematic and comprehensive evaluation of each module in the three currently representative models, including Convolutional Neural Network, Denoising Autoencoder, and Wide & Deep, which were adopted by CNNGWP, DeepAMR, and WDNN, respectively, was performed in this framework regarding module contributions in order to assemble a novel model with proper dedicated modules. Based on the whole-genome level mutations, a de novo learning method was developed to overcome the intrinsic limitations of previous models that rely on known drug resistance-associated loci. A customized DL model with the multilayer perceptron architecture was constructed and achieved a competitive performance (the mean sensitivity and specificity were 0.90 and 0.87, respectively) compared to previous ones. The new model developed was applied in an end-to-end user-friendly graphical tool named TB-DROP (TuBerculosis Drug Resistance Optimal Prediction: https://github.com/nottwy/TB-DROP), in which users only provide sequencing data and TB-DROP will complete analysis within several minutes for one sample. Our study contributes to both a new strategy of model construction and clinical application of deep learning-based drug-resistance prediction methods.

**Supplementary Information:**

The online version contains supplementary material available at 10.1186/s12864-024-10066-y.

## Introduction

Tuberculosis, caused by *Mycobacterium tuberculosis* (MTB), is a serious public health problem worldwide. According to Global Tuberculosis Report 2022 [1], 6.4 million patients were newly diagnosed with tuberculosis, among whom about 1.4 million HIV-negative patients and 187,000 HIV-positive patients died in 2021. Tuberculosis is also a high health burden in China [2]. The emergence of drug-resistant MTB has posed a severe challenge to global tuberculosis prevention and treatment. Drug resistance is traditionally diagnosed using culture-based antimicrobial susceptibility testing. However, this approach is relatively slow and expensive. Further, it has inherent inaccuracies and issues with reproducibility [3]. One critical challenge in tackling the global TB epidemic is timely diagnosis and correct treatment. Rapid molecular diagnostic tests can promote early detection and prompt treatment [4]. As drug resistance of MTB is mainly conferred by nucleotide variations in genes encoding drug targets or drug-converting enzymes [5], molecular detection of mutations can be used for quick detection and guiding treatment of drug resistant-MTB.

Currently, the sequencing technology is being used for predicting drug-susceptibility as it provides a wide range of information on mutations [6, 7]. These methods can be divided into two categories: 1) direct association (DA) method, which identifies known mutations related to drug resistance from whole genome sequencing (WGS) data, such as KvarQ [8], CASTB [9], MyKrobe Predictor TB [10], PhyResSE [11], TGS-TB [12], TBProfiler [5], and SAM-TB [13]. These tools rely heavily on the library of identified resistance-related sites and have many limitations. The missing nucleotide calls of these mutations or unknown association of mutations that affect drug resistance genes may directly lead to prediction failure. For four first-line anti-tuberculosis drugs, these problems would lead to no prediction of 4.7 − 10.2% isolates [14]. The unknown resistance mechanisms of most non-first-line drugs would lead to low prediction accuracy [15]; DA cannot model gene–gene interactions, and with the increase in MDR (multi-drug resistant) rate, the prediction performance will decrease [16]; 2) machine learning, that is, using sequencing data and drug susceptibility test (DST) data to establish a predictive model for biological phenotypes, including drug resistance. Various machine learning algorithms have been used for MTB drug resistance analysis, such as logistic regression [17], random forest [18], decision tree [19] and gradient boosting tree [20]. As a branch of machine learning, deep learning is now being widely applied for predicting biological phenotypes based on genomic mutations. Waldmann et al. [21] designed a convolutional neural network CNNGWP for genome-wide prediction. Bellot et al. [22] evaluated the performance of convolutional neural network (CNN) and multilayer perceptron (MLP) for predicting five complex human phenotypes. Regarding tuberculosis, Chen et al. [16] assessed the ability of a Wide & Deep model, which was designed for recommender system on MTB drug resistance prediction with 3,601 MTB strains and their 222 single nucleotide polymorphisms (SNPs). The Wide & Deep model outperformed existing approaches based on DA and previously reported machine learning models. Yang et al. proposed two DL-based models: denoising auto-encoder [23] and heterogeneous graph attention network [24]. Jiang et al. considered drug resistance prediction as a document classification problem and constructed a hierarchical attentive neural network model inspired by natural language processing [25]. Anna et al. divided the drug resistance prediction into multi-drug resistance prediction and single-drug resistance prediction and constructed two CNN-based models for each of them [26]. ML-based methods can establish the mapping relationship between mutations and DST data through de novo learning without prior biological knowledge and adapt to ever-increasing biological data.

As depicted above, abundant of machine learning models were adopted to predict the MTB drug resistance. Therefore, researchers started to summarize methods [27] and consider how to apply them in clinical practice [28]. Here, we take this work with concrete codes and practice, the characteristics of WDNN, DeepAMR, and CNNGWP (the representative CNN model) models were depicted comprehensively and reimplemented for benchmarking, which helped researchers evaluate the performance of each model accurately and objectively to build a foundation for improving the existing models and designing better models. The models we benchmarked were further customized and fine-tuned for developing a deep learning MTB drug resistance tool with whole genome mutations as input data. Hyperparameters and architectures of models were customized for whole genome mutations. genTB, the only available deep learning-based tool, can be easily used by clinicians [29]. However, genTB was based on known drug resistance-associated loci and was not applicable for patients harboring novel loci. Therefore, a model utilizing whole genome mutations was constructed and used in the TB-DROP (https://github.com/nottwy/TB-DROP).

This study aims to construct a customized deep learning-based model for predicting the drug resistance of MTB using whole genome mutations and bring it to clinicians through a user-friendly tool, TB-DROP. The strategies we adopted to construct our model was different from the current most widely used strategy and it would contribute to construction of a model suitable for predicting the biological phenotypes based on genotypes. The whole genome mutations were used as the input of our model and supported our de novo learning strategy without relying on a known drug resistance mutations library.

## Results

### Training and evaluation of models on our dataset

**Fig. 1:**
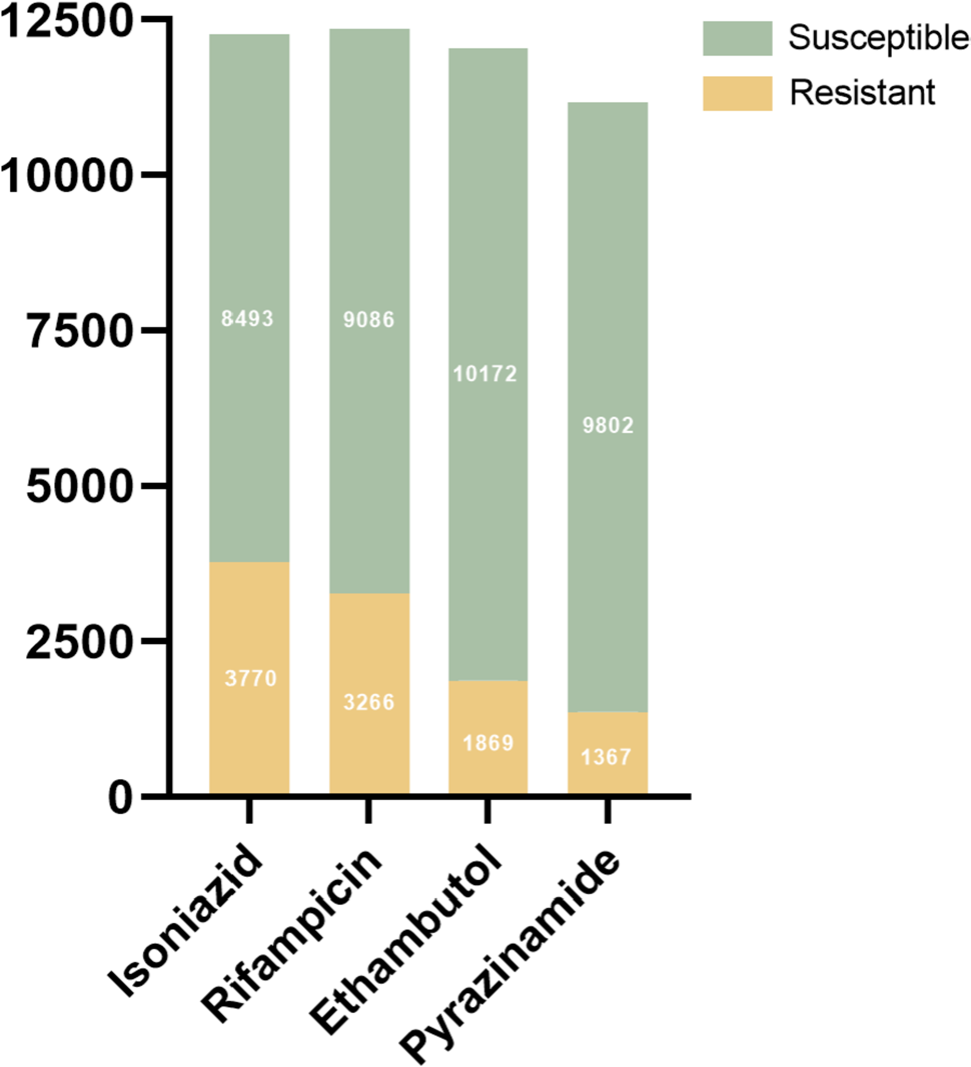
Summary of drug resistance status of all isolates

Figure 1 summarizes the phenotypes of the 12,478 MTB strains available for analysis. After variant calling and filtering, 620,169 variants are retained for drug resistance prediction. The size of the drug-sensitive samples of all types of drugs is lesser than those of the drug-resistant samples.

**Fig. 2:**
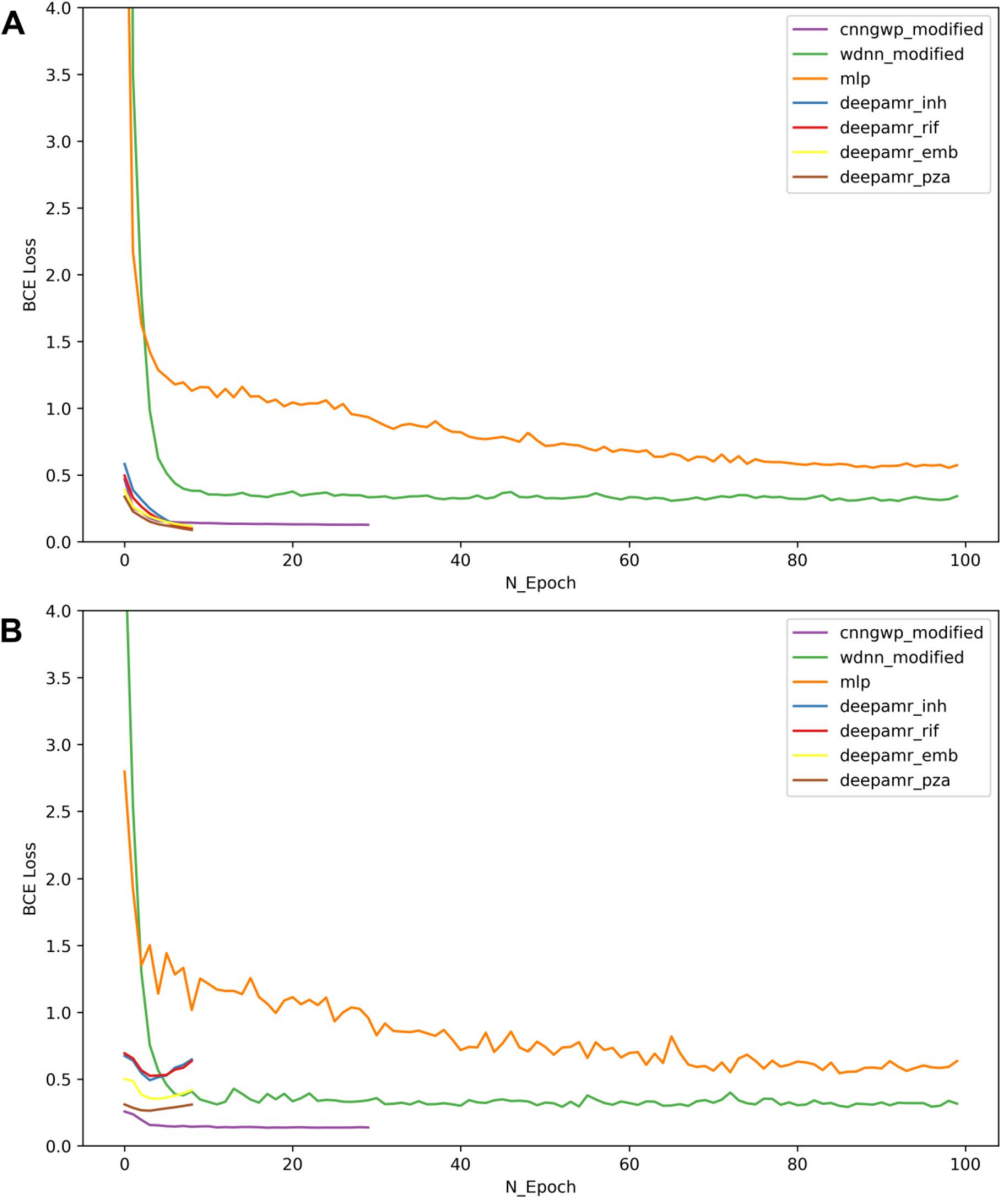
Loss curve. **A** Loss curve of training process; (**B**) Loss curve of validation process. Deepamr_inh represents the loss curve of the drug, isoniazid (INH), ethambutol (EMB), rifampicin (RIF), and pyrazinamide (PZA)

Ten-Fold (10X) cross validation with the MSSS sample split strategy was performed to measure the performance of each model. Then the mean and variance (Table 1) of AUC, and sensitivity, specificity, precision, and negative predictive rate (NPV) of the results were calculated to measure the average performance and the stability of each model. The loss curves of each model were also presented to show the training conditions of each model (Fig. 2), which could reflect whether models were reliable and were an important metric of a model (separate loss curve of each model can be found in Additional file 2). All 10 loss curves of 10X cross validation were checked for each model and found to be similar. Hence, the representative one was selected and presented here. The picture shows the loss curves of all models declined steadily and finally reached a plateau except DeepAMR. For DeepAMR, many hyperparameters were tried, but its validation loss curve was still U-shaped, indicative of overfitting.

**Table 1:** Metrics of modified four main neural network models

Model	Drug	AUC (Var)	Sensitivity (Var)	Specificity (Var)	Precision (Var)	NPV (Var)
wdnn_modified	Ethambutol	0.93 (0.000021)	0.89 (0.000332)	0.86 (0.000133)	0.55 (0.000311)	0.98 (0.000014)
	Isoniazid	0.94 (0.000020)	0.88 (0.000179)	0.92 (0.000133)	0.83 (0.000360)	0.95 (0.000029)
	Pyrazinamide	0.91 (0.000025)	0.90 (0.000400)	0.83 (0.000415)	0.42 (0.000650)	0.98 (0.000009)
	Rifampicin	0.95 (0.000015)	0.89 (0.000171)	**0.94 (0.000146)**	**0.84 (0.000598)**	0.96 (0.000018)
deepamr_modified	Ethambutol	0.89 (0.000068)	0.72 (0.000144)	**0.92 (0.000051)**	**0.62 (0.000305)**	0.95 (0.000004)
	Isoniazid	0.87 (0.000064)	0.75 (0.000215)	0.87 (0.000071)	0.72 (0.000125)	0.89 (0.000031)
	Pyrazinamide	0.89 (0.000124)	0.70 (0.000624)	**0.92 (0.000028)**	**0.56 (0.000190)**	0.96 (0.000012)
	Rifampicin	0.89 (0.000043)	0.74 (0.000314)	0.90 (0.000042)	0.74 (0.000190)	0.91 (0.000034)
cnngwp_modified	Ethambutol	**0.94 (0.000024)**	0.88 (0.000282)	0.89 (0.000261)	0.61 (0.001026)	**0.98 (0.000009)**
	Isoniazid	0.94 (0.000018)	0.87 (0.000079)	**0.92 (0.000053)**	**0.83 (0.000144)**	0.94 (0.000012)
	Pyrazinamide	**0.93 (0.000054)**	0.89 (0.000345)	0.86 (0.000040)	0.47 (0.000192)	**0.98 (0.000007)**
	Rifampicin	**0.96 (0.000007)**	**0.90 (0.000112)**	0.93 (0.000081)	0.83 (0.000285)	**0.96 (0.000012)**
MLP	Ethambutol	0.93 (0.000033)	**0.90 (0.000729)**	0.85 (0.000242)	0.52 (0.000413)	0.98 (0.000027)
	Isoniazid	**0.95 (0.000031)**	**0.88 (0.000088)**	0.90 (0.000665)	0.80 (0.001448)	**0.95 (0.000014)**
	Pyrazinamide	0.91 (0.000091)	**0.91 (0.000281)**	0.81 (0.000787)	0.41 (0.001293)	**0.98 (0.000007)**
	Rifampicin	0.95 (0.000041)	0.90 (0.000276)	0.91 (0.001085)	0.78 (0.002729)	0.96 (0.000030)

*Abbreviations: AUC* The area under the receiver operating characteristic curve, *Var* variance of values of ten folds cross-validation, Positive samples are drug resistant MTB; Negative samples are drug susceptible MTB; tp: true positive, tn: true negative, fp: false positive, fn: false negative, sensitivity: tp/(tp + fn), specificity: tn/(tn + fp), precision: tp/(tp + fp), NPV: negative predictive value, tn/(tn + fn)

The bold values indicate the highest performance values among four models. The values presented here were average values of tenfold cross validation. The values in the parenthesis are the variance of values of tenfold cross validation

### Comparison of models’ performance

The MLP-based model was chosen as the representative one and compared to other three machine learning-based models: WDNN [16], DeepAMR [23] and GBT-CRM [20]. The metrics of each model were obtained from corresponding articles, while WDNN and DeepAMR provided information regarding only sensitivity, specificity and AUC. Before comparison, two important factors that influence model metrics considerably should be introduced: the total sample size and the test size. The sample size of GBT-CRM was larger than that of ours, and fewer samples were used in the test size. Large sample sizes can train models better, and small test sample sizes would be less challenging for models. Although GBT-CRM benefited from these two factors, the difference in AUCs between it and our model was small (2.1%-5.0%) (Table 2). We expected that the difference between two models would continue to diminish when the amount of data was identical and the ratio between training and test was 1. The AUC values of all models were above 0.9 (Table 2), with the exception of WDNN’s 0.883 for PZA, which indicated that DeepAMR, GBT-CRM and TB-DROP had good and stable performance. According to metric AUC, the DeepAMR had the best performances. It is noteworthy that DeepAMR was based on the known mutations related to drug resistance and it can not work well when encountering novel mutations and unknown resistance mechanisms.

**Table 2:** The performance metrics of four machine learning models

Models	tr + v:te	Metric	RIF	INH	EMB	PZA
WDNN	3601:792	Sens	95.4%	90.3%	90.6%	75.2%
		Spe	97.9%	96.4%	85.6%	91.2%
		Acc	/	/	/	/
		NPV	/	/	/	/
		PPV	/	/	/	/
		AUC	98.2%	95.9%	92.2%	88.3%
		resis:sus	/	/	/	/
DeepAMR	7:3	Sens	94.2%	94.3%	91.5%	87.3%
		Spe	95.8%	95.7%	93.4%	90.9%
		Acc	/	/	/	/
		NPV	/	/	/	/
		PPV	/	/	/	/
		AUC	98.2%	97.7%	96.8%	94.4%
		resis:sus	/	/	/	/
GBT-CRM	8:2	Sens	88.8%	91.1%	82.8%	69.7%
		Spe	98.9%	98.8%	94.2%	96.1%
		Acc	96.2%	96.3%	92.1%	91.8%
		NPV	96.0%	95.8%	96.1%	94.2%
		PPV	96.8%	97.4%	75.6%	78.0%
		AUC	97.9%	96.7%	95.8%	95.5%
		resis:sus	4462/12045	5215/11207	2576/12254	1813/10155
TB-DROP	7:3	Sens	89.9%	88.3%	90.4%	90.7%
		Spe	90.6%	90.0%	84.9%	81.5%
		Acc	90.4%	89.5%	85.8%	82.6%
		NPV	96.2%	94.5%	98.0%	98.4%
		PPV	77.9%	80.0%	52.4%	41.0%
		AUC	95.4%	94.6%	93.2%	90.5%
		resis:sus	3266/9086	3770/8493	1869/10172	1367/9802

The values that were not reported in the models’ articles are indicated as”/”;”tr + v:te”: train + validation: test; Sens: Sensitivity; Spe: Specificity; Acc: accuracy; NPV: Negative Predictive Value (tn/(tn + fn)); PPV: Positive Predictive Value (tp/(tp + fp))

The values of the other metrics were influenced by the choice of thresholds distinguishing drug-resistant isolates and drug-susceptible isolates. They were comparable only to a certain extent. Our model paid more attention to the sensitivity of drug-resistant isolates and NPV of drug-susceptible isolates, as high sensitivity ensured that fewer drug-resistant isolates were predicted as drug-susceptible, while the high NPV ensured that antimicrobial drugs used to treat patients may work. For the drugs of RIF, EMB, and PZA, our models performed better than the GBT-CRM model in terms of metric sensitivity and NPV (Table 2). Regarding metric sensitivity, the DeepAMR and our models were stable at over 85%, while the WDNN model only achieved 75% in the drug PZA (Table 2). All these results indicated that de novo drug resistance prediction based on deep learning model utilizing whole genome mutations was as competitive as previous models and could deliver a better drug resistance predicting ability than the deep learning models based on the known drug resistance genes and machine learning models based on whole genome mutations.

### TB-DROP: MTB Drug Resistance Optimal Predictor

**Fig. 3:**
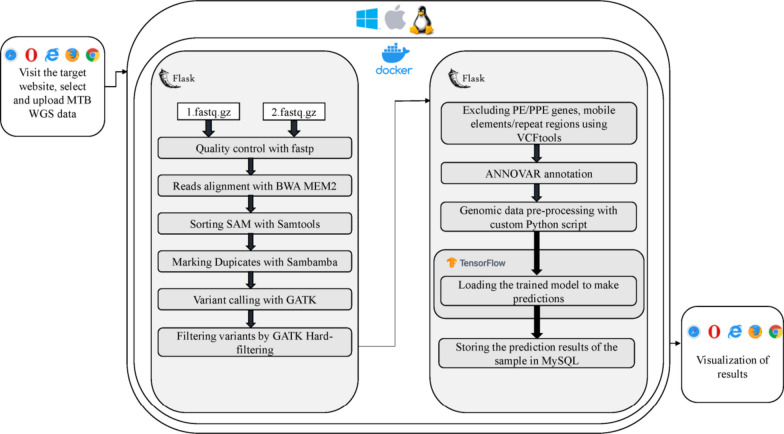
The workflow of TB-DROP

**Fig. 4:**
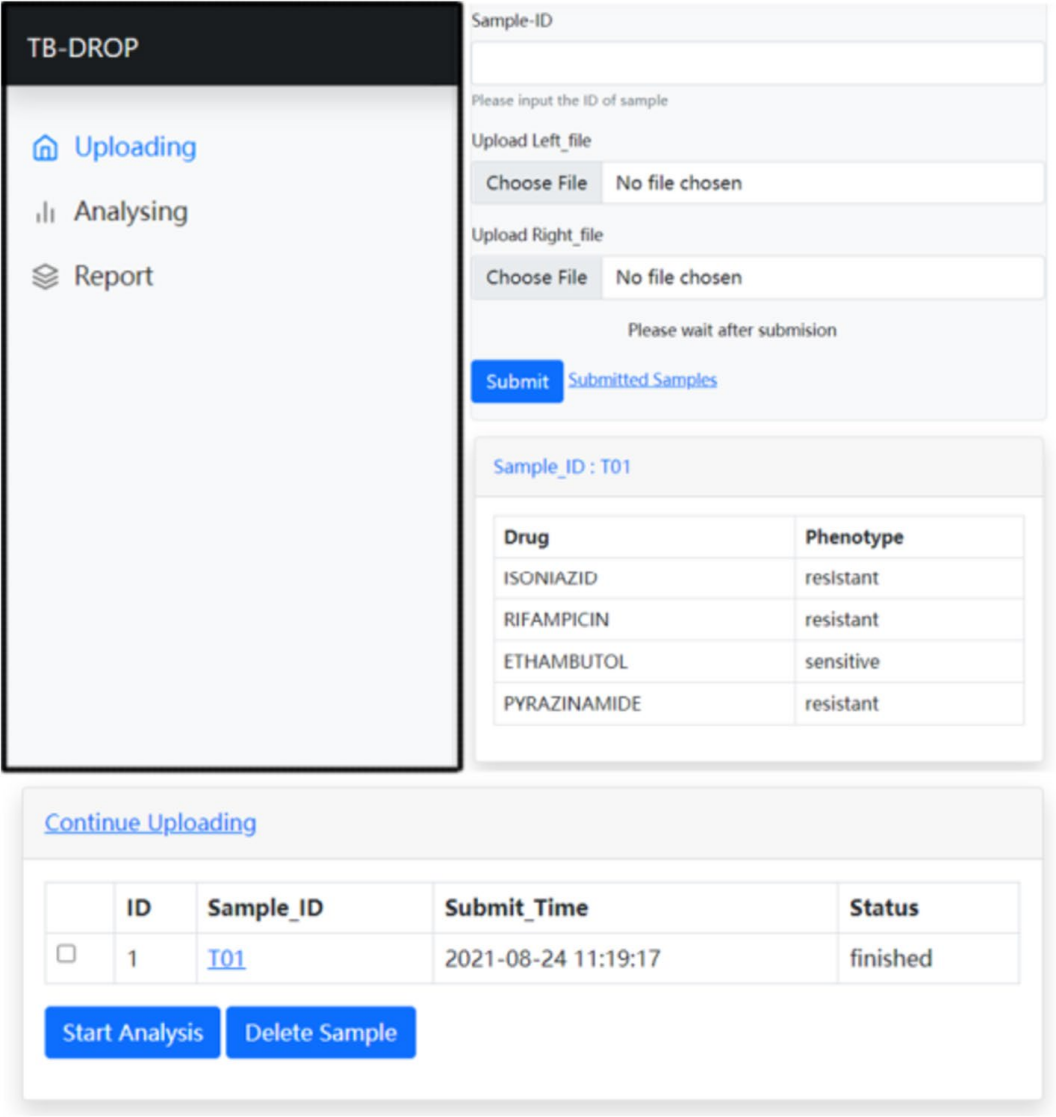
TB-DROP user interface

The trained deep learning model and the variant calling pipeline were used in a docker environment. The workflow and user interface of TB-DROP are shown in Figs. 3 and 4. In the TB-DROP interface, users only need to perform a simple two-step operation to obtain drug resistance status of MTB: “Uploading” the sequencing data in the fastq format and click “Start Analysis”. The whole analysis costs only < 20 min for each sample on a computer with an AMD Ryzen 5 2600 Six-core Processor with 16G RAM. For samples that have been analyzed, the prediction result will be presented while users click the Sample_ID.

## Discussion

Laboratory mislabeling of the drug resistance status of MTB should be excluded. A reliable standard for removing laboratory mislabeling involved removal of isolates phenotypes of which were discordant with the genotypes. For example, one isolate was recorded as susceptible but harbored high-level resistance mutations. One main aims of this study was to evaluate the potential of the neural network for predicting de novo drug resistance by utilizing the whole genome mutations. Therefore, only first-line drugs with large sample size (more than that of second-line drugs) were evaluated in this study. It is reasonable to infer that the performance of DL models is similar here for the second-line drugs when their sample sizes achieve similar levels.

The three models used in this study were not designed specifically for predicting phenotypes according to genotypes. CNN, the architecture of which was inspired by the human visual system, was proposed by LeCun et al. [30] for recognizing handwritten zip code. WDNN [31] was developed for constructing the recommender system, the original inputs of which were user and contextual information, and the desired output was relevant items that users might be interested in. The successful application of these models on biological phenotypes only reflected that there were similar relationships between inputs and outputs. An architecture designed specifically according to biological genotype–phenotype relationships was in demand [22]. The first step toward realization of this goal was to evaluate the performances of different architectures objectively. Nevertheless, due to the complexity of deep learning neural architectures and MTB drug resistance, a comprehensive benchmark of these models was not performed yet, which prevents researchers from the development of the most suitable model and enhancement of prediction performance [32].

The relatively low PPV for predicting the EMB and PZA drug resistance (0.524 for EMB and 0.410 for PZA) largely came from the imbalanced dataset (Table 2), where the ratio of positive to negative samples was far from 1 (0.184 for EMB and 0.139 for PZA). When the ratio was 1, the PPV was around 0.85 (assuming the numbers of positive and negative samples were both 2,000, and the sensitivity and specificity did not change, the PPVs were 0.857 and 0.831, respectively). Therefore, the PPV does not influence the sensitivity, and most of the patients carrying drug resistant MTB, including EMB and PZA, should be detected correctly using our predication tool.

Several methods can be used to improve the performance of deep learning models. First, collection of reliable data is critical. Currently, the input features were encoded as 0 and 1, where 0 represented no mutation and 1 represented a mutation, although there were other commonly used representation methods, such as “012”, where 0 represents no mutation, 1 represents heterozygous genotype, and 2 represents homozygous alternative genotype and “one-hot encoding”. Further evaluation is required to evaluate the suitability. Each model evaluated in this study had its specific preprocessing steps. However, we have not evaluated the performance of all these preprocessing methods. In addition, correct conclusion can only be obtained from the correct combination of all components, from input representation to model architecture and hyperparameters. Unsuitable combination may affect the function of components. A model architecture that was more suitable for genomic mutations and prediction of biological phenotypes can be designed referring to the following perspectives: 1) determining the functions of various types of mutations in the genome, including the relationship among mutations and that between mutations and phenotypes [33], and then designing an architecture that can represent such relationships; 2) multi-task or single-task. The multitask neural network updated the weights of the network according to the total loss of all tasks and the single-task neural network learned the weights for a specific drug. The multitask neural network can learn from all labels and hence had more samples. However, different labels may conflict with each other, and lead to wrong update of the weights of the neural network and finally perturb the metrics of the model. The bias of the final layer of the MLP model was updated weirdly, which might be caused by the multitask architecture; 3) the ideal solution for finding the best hyperparameters was to be able to traverse all hyperparameter combinations and then consider the optimal hyperparameter combination. However, this will require considerable amount of computational resources and time. One main aims of this study was to evaluate each module of multiple representative models and to assemble a new model based on the contribution of each module. In future, we will improve the hyperparameter tuning strategy; 4) in addition to the three representative deep learning models used here, models of natural language processing can also be applied to deal with drug resistance prediction, if we consider the whole genome mutations as a document and the resistant and the susceptible phenotypes as two types of the document.

The modules and the learning processes of any existing models were proposed for their own aims. Therefore, we need to deepen our understanding of the mechanism of how genotypes determine phenotypes and reveal the mathematical functions and learning processes that can truly characterize the mechanism. In this way, we can change from simply borrowing modules from existing models to proposing really suitable modules and learning processes. As a tool positioned for use in clinical practice, TB-DROP also requires continuous accumulation of experience and update of models to deal with problems arising in practice.

### Implementation

#### Phenotypic and sequencing data

The datasets were obtained from two previously published studies and consist of 12,478 isolates with WGS data and phenotypic DST data [14, 34]. The SRA accession numbers of all raw datasets are listed in Additional file 3. The phenotype data included resistance status for four first-line drugs (rifampicin, isoniazid, pyrazinamide, and ethambutol). Phenotypic data were classified as resistant, susceptible, or unknown.

#### Variant calling

We used fastp 0.20.1 [35] to clean the raw sequencing data. The cleaned reads were mapped to the H37Rv reference genome using BWA-MEM 0.7.17 [36], and SNPs, insertions, and deletions (InDels) were called using GATK 4.1.7.0 [37]. Variant annotation was performed using ANNOVAR [38], and variants annotated as synonymous mutations were not included in our analysis. SNPs and InDels in PE (Pro-Glu) or PPE (Pro-Pro-Glu) genes, mobile elements, and repeat regions were excluded using VCFtools 0.1.16 [39].

#### Building the predictor sets of features

The features used for prediction were classified into two groups. In one group, each mutation in the genome was used as the predictive feature. The presence of a mutation in the isolate was represented by a binary variable, with 1 indicating the presence of the mutation and 0 indicating its absence. In the other group, to reduce the feature dimension, we used 100-bp windows to divide the entire genome into 44,116 regions, and the number of mutations in each region was considered the predictor.

#### Designing and training the TB-DROP model

Both designing the novel models and comprehensively comparing the existing methods are essential for developing an efficient neural network for MTB drug resistance. Multiple neural networks have been used to predict biological phenotypes, including MTB drug resistance. To use the best neural network in our tool, four architectures were summarized, reimplemented, optimized, and compared.

First, we summarized the features, advantages, and disadvantages of each model, with the intention of mainly targeting neural network designers. The comprehensive and in-depth summary may facilitate the construction of more suitable models. Next, we reimplemented WDNN, DeepAMR, and CNNGWP in the same framework according to their published source codes. The same framework guaranteed that each model can be depicted systematically. In this way, we could clearly determine the modules to be used for each model. In addition, it was convenient to append a module to a model at the right place. The reimplemented version of each model was evaluated with the datasets available with the source codes to prove that we did restore the model to a certain extent.

Each model was optimized to accommodate the condition that the whole genome mutations were used as inputs, during which the advantages of each model were retained and the defects overcome. Our dataset was used as the input of each model, which was optimized according to its training/validation loss curves and metrics on the validation dataset. The hyperparameters were tuned according to their functions and the depth up to which learning models gain satisfied generalization. For all models, the weight of the neural network with the lowest validation loss value during the training process was used in the final model.

Finally, the optimized models were evaluated on the test dataset, and the model with the best performance was selected as the representative model and compared with other MTB drug resistance prediction methods. The best model was used in our tool, TB-DROP. tensorflow-gpu (2.3.1) was used.

### TB-DROP graphical user interface

TB-DROP is based on Docker (https://www.docker.com/) to enable a new and promising virtualization strategy that provides the advantage of being platform-agnostic due to its configuration of containers. Containers can be consistently interchanged and used in different computing environments, irrespective of the differences in user hardware and/or operating systems. These features of the containers ensure replicability and reproducibility of data analyses across different facilities [40].

TB-DROP is an automated, easy-to-use, and web-based GUI tool deposited at github (https://github.com/nottwy/TB-DROP). During development, the bioinformatics pipeline, trained deep learning model, and user-interface in a custom docker image were used, and the characteristics of the docker were utilized to develop tools compatible with the cross-platforms, including Windows, Linux, and MacOS. The resistance results can be visualized on a browser directly. Users need to upload the sequencing data on the webpage and initiate the analysis. The result of drug resistance is usually returned in a few minutes.

### Summarization of MTB-related and phenotype-related DL models

Each model has its own advantages and unique features, as well as limitations. Extensively learning advantages of each model, and then avoiding shortcomings in their designs will assist in designing better models. The characteristics of each model are shown in Table 3. The wide and deep architecture enables WDNN to consider additive effects and interactions between mutations simultaneously [16, 31]. WDNN utilized an alpha value as the class weight to increase the weight of the class, the sample size of which was smaller.

$$\alpha =1-\frac{n1}{n1+n2}$$

where n1 was the number of drug-resistant MTB and n2 was the number of drug-susceptible MTB. The number of MTB, the resistance status of which was missing, was not considered. The custom loss function was a class-weight binary cross entropy:

$$loss=\sum_i^{i=n}\sum_j^{j=m}-\alpha\times P_{rtrue,ij}\times log\left(P_{rpred,ij}\right)-\left(1-\alpha\right)\times\left(1-P_{rtrue,ij}\right)\times log\left(1-P_{rpred,ij}\right)$$

where n was the total types of drugs, m represents the number of isolates with resistance status for each drug, P_rtrue,ij_ indicated the true probability for the i-th drug and j-th MTB being resistant (1.0 for resistant MTB and 0.0 for susceptible MTB). P_rpred,ij_ indicated the predicted probability for the i-th drug and j-th MTB being resistant. Since WDNN was a multi-task model that had only one loss for all drugs, ∑∑ was used to add up all class-weight binary cross entropy of each sample.

**Table 3:** Summary of the four main neural network models

Model	WDNN	DeepAMR	CNNGWP	TB-DROP
Features	1. Wide: Memorization 2. Deep: generalization 3. Custom loss and metrics functions 4. Remove rare variants	1. Denoising autoencoder 2. Cyclical learning rate	1. CNN 2. Normalization of input 3. MAF cleaning	Fully connected
Advantages	1. Allow missing labels 2. Batch Normalization	1. Non-linear dimension reduction 2. Quicker converge	1. Convolution: sparse interactions, parameter sharing and equivariant representations 2. Pooling: approximately invariant to small change of the input	Explore all interactions
Drawbacks	Too many neurons	Not allow missing labels	Test datasets were also used as validation datasets	Too many neurons
Regularization	1. Multi-task learning 2. Dropout 3. Parameter norm penalty	1. Multi-task learning 2. Early stopping	1. Single-task learning 2. Model averaged ensemble predictions	Multi-task learning
Hyperparameter tuning	Bayesian Optimization	Grid search	Bayesian Optimization	Manual search
Speed	~ 3 s/epoch	Pretrain: ~ 76 s/epoch Train: ~ 110 s/epoch	~ 40 s/epoch	~ 10 s/epoch
Threshold	max(tpr + tnr)	max(tpr-tnr)	max(tpr + tnr)	max(tpr + tnr)
CV strategy	KFold	MSSS	MSSS	MSSS
Software^a^	No	No	No	Yes

*Abbreviations: MAF* Minor Allele Frequency, *CNN* Convolutional Neural Network, *KFold* sklearn.model_selection.KFold, *CV* Cross Validation, *MSSS* python package, iterstrat.ml_stratifiers.MultilabelStratifiedShuffleSplit, *tpr* true positive rate, *tnr* true negative rate,

^a^Whether providing softwares that could be used

In addition to predicting the resistance to individual drugs, DeepAMR also predicted the drug resistance of MDR-TB (multi-drug resistant *Mycobacterium tuberculosis*) and PANS-TB (MTB that is susceptible to all four first-line drugs, isoniazid (INH), rifampicin (RIF), ethambutol (EMB), and pyrazinamide(PZA)). The further classification of drug resistance phenotypes remind us that the mechanism controlling drug resistance might change if MTB developed from single-drug resistance to multi-drug resistance. Manzour et al. [41] reported that mutations in *kat*G315 appeared more frequently in MDR-TB and that mutations in the *inh*A promoter appeared more frequently in single-drug resistant MTB. Sintchenko et al. [42] observed that mutations in *rpo*B existed both in RIF-resistant MTB and RIF-susceptible MTB. Regarding the model architecture, DeepAMR used the denoising autoencoder, which rendered the model more robust when genotypes were missing and sequencing error was present. Furthermore, the dimension reduction achieved using the autoencoder can considerably reduce the amount of calculation.

Furthermore, a better sample splitting strategy, multilabel stratified shuffle split (MSSS) [43] was used by DeepAMR than WDNN’s naive KFold (provided in sklearn), which does not consider group information. The drawback of KFold was that random splitting of samples could lead to a split (train/test) where the train group or the test group did not contain all categories for one or more labels in a multi-label condition. This would lead to the failure of training if it happens in the training dataset, as some categories would be missing. So we performed it in the test dataset to assess the failure of calculating some metrics (i.e., missing positive samples would lead to the failure of calculation of sensitivity because sensitivity equals to “true positive / all positive samples”).

Using SNPs from the whole genome, Waldmann et al. [21] attempted to apply CNN to predict quantitative traits. CNN has many advantages in predicting phenotypes using genomic mutations. The small convolutional kernels can capture local signals out of the whole genome that might be related to drug resistance and save computational resource at the same time. The pooling layer can make the model robust when there were little changes in the genome.

The MLP is a quintessential neural network model which was proposed long time ago. A typical MLP consists of an input layer, an output layer, and many hidden layers. These layers are fully connected with each other, because of which MLP requires many computational resources. The reason for choosing this model was: (1) it is initially inspired by WDNN. If we modified the structure of the WDNN model and removed the wide part of it, the rest of the WDNN model was a DNN (or MLP) model; (2) it has the most basic architecture and we should evaluate its performance before we try other more complex architectures; (3) many researchers have started to focus on MLP again and have proposed many excellent architectures to improve its performance [44]. The final result was that MLP had performed best before we tried new advanced MLP models.

### Reimplementation of deep learning models

Although all the models evaluated in this study were implemented with keras (with tensorflow as their backend engine), they were implemented with tensorflow 1.X (WDNN, DeepAMR and CNNGWP) or R version (CNNGWP). Tensorflow 1.X is deprecated and the grammar changed considerably in tensorflow 2.X. Models implemented using different languages (R and Python tensorflow) increased the difficulty of utilization and comparison. Therefore, reimplementation is necessary and all models were reimplemented with Python in the tensorflow 2.X environment. As we will utilize the good design of each published model and make an objective comparison to construct a better MTB drug resistance predicting model, and provide guidelines for optimizing the neural network-based phenotype predictors in the future, we implemented all models in the same standard framework and ensured that all modules and parameters were consistent with the original model. We first carefully inspected the source code of each model and implemented each model in strict accordance with the source code. Next, these models were used in the same framework to ensure that the modules used by each model were the same and that the execution order was the same. Finally, we tested the performance of our reimplemented versions and the original source code versions on the dataset accompanying each source code to measure the consistency between the reimplemented and the original models. The outputs of the source codes of each model were used as the standard answers.

The outputs of our implementation were compared with standard answers (Table 4) and the datasets used for each model were introduced below. WDNN’s author provided both the training and test datasets. The training datasets consisted of 3,601 isolates and 6,483 SNPs, and the test dataset consisted of 792 isolates and 222 SNPs. In the WDNN’s source codes, it predicted MTB drug resistance of 11 drugs. Area under the ROC curve (AUC) and AUC precision-recall (PR) were chosen as the metrics. Therefore, the difference of sum of AUC between our reimplemented version and the original version was chosen as the measurement of how well we reimplemented the model. The authors of DeepAMR provided a dataset consisting of 8,388 isolates and 5,823 SNPs. It only predicted MTB drug resistance of four drugs. Sensitivity, specificity, AUC, and F1 scores were selected as its metrics. Here, the difference of sum of AUC between our reimplemented version and the original version was also chosen as the measurement of how well we reimplemented the model. In the publication of CNNGWP, two datasets, a simulated and a real dataset, were used to evaluate the performance of the CNNGWP model. The real dataset is not accessible. Therefore, only the performance on the simulated dataset was compared. Input introduction: 3,226 samples and 9,723 SNPs. The input representations of 0, 1, and 2 indicated lower homozygote, heterozygote, and upper homozygote. The prediction target here was a continuous quantitative trait, and the mean squared error (MSE) of the test dataset was used to evaluate the performance of CNNGWP. The sizes for training and testing datasets were 2,326 and 900. Using the best hyperparameters provided in the article, the MSE on the test dataset was 63.04 in our implementation, which was almost similar to that reported in this study (62.34). The comparison is presented in Table 4, and we noticed that the performances of our reimplemented version and the original version were similar. Therefore, our reimplemented version represented the original one.

**Table 4:** The difference of metrics between the original models and the reimplementation version

Model	Metrics	Original	Reimplementation	Difference (%)
WDNN	Sum of AUC	10.50	10.50	0
DeepAMR	Sum of AUC	3.75	3.87	3.20
CNNGWP	MSE	62.34	63.04	1.12

The column ‘difference’ is calculated as (value of reimplementation – value of original) / value of original. AUC stands for “Area under the ROC Curve”; MSE stands for Mean Squared Error

### Construction of TB-DROP deep learning models

**Fig. 5:**
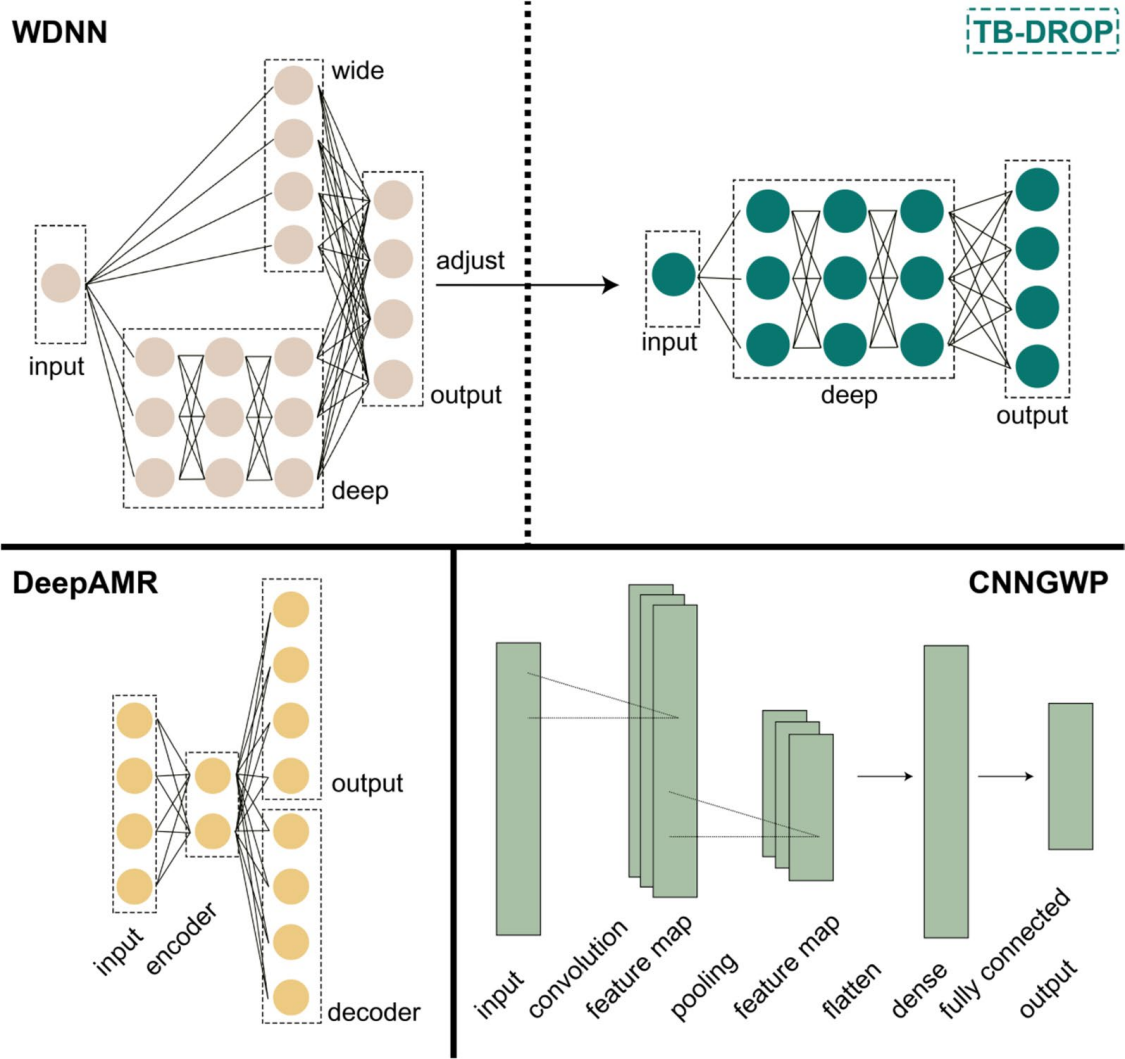
The representative architectures of four models, including the TB-DROP. The upper-left panel is the model architecture of WDNN, which comprises two parts: wide part and deep part. The model architecture implemented in TB-DROP (upper-right) is a deep neural network (DNN). The bottom-left panel is the model architecture of DeepAMR, which comprises encoder, decoder, and output layers. The model architecture of CNNGWP in the bottom-right panel is a classic convolutional neural network which consists of a convolutional layer and a pooling layer

As we switched to using whole-genome mutations as input, the amount of layer weights was large. Therefore, the model architecture and hyperparameter tuning strategy adopted were intended consider the published models as the starting point and then further tune them according to the problems encountered. The representative architectures and characteristics of the four models are presented in Fig. 5. Capturing the core features of each model and the essential differences between the models when putting them together was easier. More details regarding hyperparameter tuning are presented in Additional file 1.

The goal of the original design of WDNN was to enable the final classification layer to learn (1) directly from the input data (the wide part) and (2) the highly abstract features after refining through multiple neural layers (the deep part) simultaneously (Fig. 5). However, when the input data changed from the mutations residing in the drug resistance-related genes to mutations in the whole genome, the number of mutations increased considerably. Most of the newly added mutations must not be related to drug resistance. Therefore, the model training faced more computational pressure, and at the same time, the final output layer found it challenging to learn the mapping relationship between whole genome mutations and the drug resistance phenotypes. DeepAMR consisted of two parts: (1) The model will train a denoising autoencoder first, the input and output of which were identical. (2) The highly compressed and dimension-reduced encoded features obtained from part one would be fully connected to the output layer to predict the drug resistance of MTB (Fig. 5). The main features of CNN-based model were the convolution and pooling layers (Fig. 5). A convolution layer could learn the interaction between mutations and save more computation than a fully connected layer. A pooling layer can increase the model’s ability of resisting noise and save computation. The model used in TB-DROP, a MLP (Fig. 5), was constructed finally based on the observation and summarization during the adjustment of the three published models. As analyzed above, we found that the wide part in the original design of WDNN is no longer suitable for our scenario. Therefore, the wide part of WDNN was removed from the architecture of WDNN and the model became a traditional MLP model. The performance of the model did not change, indicating the wide part that WDNN contributed negligibly.

## Conclusion

The deep learning training framework developed in this study contributes substantially to the in-depth understanding of the characteristics of the models, as well as the standardization and optimization of the training process. The three representative models were summarized and benchmarked systematically and comprehensively using this framework to discover the strengths and weaknesses of these modules, which provided a reliable basis for researchers who aim to develop more effiecient deep learning-based models. The de novo MTB drug resistance prediction tool TB-DROP developed to overcome the previous limitations from novel mutations and second-line drugs and the rarely reported drug-resistance genes. The small variance (the stable performance in 10 × cross validation) was a symbol of stability and convergent loss curve, which indicated a model was well-trained. These works guarantee the reliability of the model provided in TB-DROP. The development of TB-DROP cleared the barriers for clinicians in applying deep learning models, as well as laid the foundation for the application of highly efficient models in the clinic in the future.

## Data availability and requirements

**Project name:** TB-DROP.

**Project home page:** https://github.com/nottwy/TB-DROP

**Operating system(s):** Linux, Windows and macOS.

**Programming language:** Python.

**Other requirements:** Docker.

**License:** Apache 2.0.

**Any restrictions to use by non-academics:** None.

## Supplementary Information

**Additional file 1.** Supplementary Materials.

**Additional file 2.** Separate loss curves for each model. X-axis represents epochs and Y-axis represents loss values. Because each model converges after different epochs, the ranges of X-axis of each model are different from each other. This file corresponds to Fig. 3 in the manuscript. The pictures below are used to show more details of training process so they are presented separately.

**Additional file 3: Supplementary Table 1.** SRA accession numbers.

## Data Availability

All data generated or analyzed during this study are included in this published article and its supplementary information files.

## References

[1] WHO. Global Tuberculosis Report. Geneva: World Health Organization; 2022. p. 2.

[2] Fa L, Xu C, Cheng J, Zhang H. Acceptability of tuberculosis preventive treatment strategies among healthcare workers using an online survey—China, 2021. China CDC Weekly. 2022;4(11):211–5. 35433078 10.46234/ccdcw2022.050 PMC9005477

[3] Farhat MR, Sultana R, Iartchouk O, Bozeman S, Galagan J, Sisk P, et al. Genetic determinants of drug resistance in mycobacterium tuberculosis and their diagnostic value. Am J Respir Crit Care Med. 2016;194(5):621–30. 26910495 10.1164/rccm.201510-2091OC PMC5027209

[4] Yang Y, Niehaus KE, Walker TM, Iqbal Z, Walker AS, Wilson DJ, et al. Machine learning for classifying tuberculosis drug-resistance from DNA sequencing data. Bioinformatics. 2018;34(10):1666–71. 29240876 10.1093/bioinformatics/btx801 PMC5946815

[5] Phelan JE, O’Sullivan DM, Machado D, Ramos J, Oppong YE, Campino S, et al. Integrating informatics tools and portable sequencing technology for rapid detection of resistance to anti-tuberculous drugs. Genome Med. 2019;11(1):41. 31234910 10.1186/s13073-019-0650-x PMC6591855

[6] Coll F, Phelan J, Hill-Cawthorne GA, Nair MB, Mallard K, Ali S, et al. Genome-wide analysis of multi- and extensively drug-resistant Mycobacterium tuberculosis. Nat Genet. 2018;50(2):307–16. 29358649 10.1038/s41588-017-0029-0

[7] Dheda K, Gumbo T, Maartens G, Dooley KE, McNerney R, Murray M, et al. The epidemiology, pathogenesis, transmission, diagnosis, and management of multidrug-resistant, extensively drug-resistant, and incurable tuberculosis. Lancet Respir Med. 2017;5(4):291–360. 10.1016/S2213-2600(17)30079-6 28344011

[8] Steiner A, Stucki D, Coscolla M, Borrell S, Gagneux S. KvarQ: targeted and direct variant calling from fastq reads of bacterial genomes. BMC Genomics. 2014;15(1):1–12. 25297886 10.1186/1471-2164-15-881 PMC4197298

[9] Iwai H, Kato-Miyazawa M, Kirikae T, Miyoshi-Akiyama T. CASTB (the comprehensive analysis server for the Mycobacterium tuberculosis complex): a publicly accessible web server for epidemiological analyses, drug-resistance prediction and phylogenetic comparison of clinical isolates. Tuberculosis (Edinb). 2015;95(6):843–4. 26542225 10.1016/j.tube.2015.09.002

[10] Bradley P, Gordon NC, Walker TM, Dunn L, Heys S, Huang B, et al. Rapid antibiotic-resistance predictions from genome sequence data for Staphylococcus aureus and Mycobacterium tuberculosis. Nat Commun. 2015;6:10063. 26686880 10.1038/ncomms10063 PMC4703848

[11] Feuerriegel S, Schleusener V, Beckert P, Kohl TA, Miotto P, Cirillo DM, et al. PhyResSE: a web tool delineating Mycobacterium tuberculosis antibiotic resistance and lineage from whole-genome sequencing data. J Clin Microbiol. 2015;53(6):1908–14. 25854485 10.1128/JCM.00025-15 PMC4432036

[12] Sekizuka T, Yamashita A, Murase Y, Iwamoto T, Mitarai S, Kato S, et al. TGS-TB: total genotyping solution for Mycobacterium tuberculosis using short-read whole-genome sequencing. Plos One. 2015;10(11): e0142951. 26565975 10.1371/journal.pone.0142951 PMC4643978

[13] Yang T, Gan M, Liu Q, Liang W, Tang Q, Luo G, et al. SAM-TB: a whole genome sequencing data analysis website for detection of Mycobacterium tuberculosis drug resistance and transmission. Brief Bioinform. 2022;23(2):bbac030. 10.1093/bib/bbac030. 35211720 10.1093/bib/bbac030 PMC8921607

[14] Consortium CR, the GP, Allix-Beguec C, Arandjelovic I, Bi L, Beckert P, et al. Prediction of susceptibility to first-line tuberculosis drugs by DNA sequencing. N Engl J Med. 2018;379(15):1403–15. 30280646 10.1056/NEJMoa1800474 PMC6121966

[15] Schleusener V, Köser CU, Beckert P, Niemann S, Feuerriegel S. Mycobacterium tuberculosis resistance prediction and lineage classification from genome sequencing: comparison of automated analysis tools. Sci Rep. 2017;7(1):1–9. 28425484 10.1038/srep46327 PMC7365310

[16] Chen ML, Doddi A, Royer J, Freschi L, Schito M, Ezewudo M, et al. Beyond multidrug resistance: Leveraging rare variants with machine and statistical learning models in Mycobacterium tuberculosis resistance prediction. EBioMedicine. 2019;43:356–69. 31047860 10.1016/j.ebiom.2019.04.016 PMC6557804

[17] Zhang H, Li D, Zhao L, Fleming J, Lin N, Wang T, et al. Genome sequencing of 161 Mycobacterium tuberculosis isolates from China identifies genes and intergenic regions associated with drug resistance. Nat Genet. 2013;45(10):1255–60. 23995137 10.1038/ng.2735

[18] Kouchaki S, Yang Y, Lachapelle A, Walker TM, Walker AS, Peto TE, et al. Multi-label random forest model for tuberculosis drug resistance classification and mutation ranking. Front Microbiol. 2020;11:667. 32390972 10.3389/fmicb.2020.00667 PMC7188832

[19] Deelder W, Napier G, Campino S, Palla L, Phelan J, Clark TG. A modified decision tree approach to improve the prediction and mutation discovery for drug resistance in Mycobacterium tuberculosis. BMC Genomics. 2022;23(1):1–7. 35016609 10.1186/s12864-022-08291-4 PMC8753810

[20] Deelder W, Christakoudi S, Phelan J, Benavente ED, Campino S, McNerney R, et al. Machine learning predicts accurately mycobacterium tuberculosis drug resistance from whole genome sequencing data. Front Genet. 2019;10:922. 31616478 10.3389/fgene.2019.00922 PMC6775242

[21] Waldmann P, Pfeiffer C, Meszaros G. Sparse convolutional neural networks for genome-wide prediction. Front Genet. 2020;11:25. 32117441 10.3389/fgene.2020.00025 PMC7029737

[22] Bellot P, de Los CG, Perez-Enciso M. Can deep learning improve genomic prediction of complex human traits? Genetics. 2018;210(3):809–19. 30171033 10.1534/genetics.118.301298 PMC6218236

[23] Yang Y, Walker TM, Walker AS, Wilson DJ, Peto TEA, Crook DW, et al. DeepAMR for predicting co-occurrent resistance of Mycobacterium tuberculosis. Bioinformatics. 2019;35(18):3240–9. 30689732 10.1093/bioinformatics/btz067 PMC6748723

[24] Yang Y, Walker TM, Kouchaki S, Wang C, Peto TE, Crook DW, et al. An end-to-end heterogeneous graph attention network for Mycobacterium tuberculosis drug-resistance prediction. Brief Bioinform. 2021;22(6):bbab299. 10.1093/bib/bbab299. 34414415 10.1093/bib/bbab299 PMC8575050

[25] Jiang Z, Lu Y, Liu Z, Wu W, Xu X, Dinnyés A, et al. Drug resistance prediction and resistance genes identification in Mycobacterium tuberculosis based on a hierarchical attentive neural network utilizing genome-wide variants. Brief Bioinform. 2022;23(3):bbac041. 10.1093/bib/bbac041. 35325021 10.1093/bib/bbac041

[26] Green AG, Yoon CH, Chen ML, Ektefaie Y, Fina M, Freschi L, et al. A convolutional neural network highlights mutations relevant to antimicrobial resistance in Mycobacterium tuberculosis. Nat Commun. 2022;13(1):3817. 35780211 10.1038/s41467-022-31236-0 PMC9250494

[27] Singh M, Pujar GV, Kumar SA, Bhagyalalitha M, Akshatha HS, Abuhaija B, et al. Evolution of machine learning in tuberculosis diagnosis: a review of deep learning-based medical applications. Electronics. 2022;11(17):2634.

[28] Kim JI, Maguire F, Tsang KK, Gouliouris T, Peacock SJ, McAllister TA, et al. Machine learning for antimicrobial resistance prediction: current practice, limitations, and clinical perspective. Clin Microbiol Rev. 2022;35(3):e00179–e221. 35612324 10.1128/cmr.00179-21 PMC9491192

[29] Gröschel MI, Owens M, Freschi L, Vargas R, Marin MG, Phelan J, et al. GenTB: A user-friendly genome-based predictor for tuberculosis resistance powered by machine learning. Genome Med. 2021;13(1):1–14. 34461978 10.1186/s13073-021-00953-4 PMC8407037

[30] LeCun Y, Boser B, Denker JS, Henderson D, Howard RE, Hubbard W, et al. Backpropagation applied to handwritten zip code recognition. Neural Comput. 1989;1(4):541–51.

[31] Cheng H-T, Koc L, Harmsen J, Shaked T, Chandra T, Aradhye H, et al., editors. Wide & deep learning for recommender systems. Proceedings of the 1st workshop on deep learning for recommender systems. New York; 2016.

[32] Weber LM, Saelens W, Cannoodt R, Soneson C, Hapfelmeier A, Gardner PP, et al. Essential guidelines for computational method benchmarking. Genome Biol. 2019;20(1):1–12. 31221194 10.1186/s13059-019-1738-8 PMC6584985

[33] Szydlowski M, Paczynska P. QTLMAS 2010: simulated dataset. BMC Proc. 2011;5(Suppl 3):S3. 21624173 10.1186/1753-6561-5-S3-S3 PMC3103202

[34] Walker TM, Kohl TA, Omar SV, Hedge J, Elias CDO, Bradley P, et al. Whole-genome sequencing for prediction of Mycobacterium tuberculosis drug susceptibility and resistance: a retrospective cohort study. Lancet Infect Dis. 2015;15(10):1193–202. 26116186 10.1016/S1473-3099(15)00062-6 PMC4579482

[35] Chen S, Zhou Y, Chen Y, Gu J. fastp: an ultra-fast all-in-one FASTQ preprocessor. Bioinformatics. 2018;34(17):i884–90. 30423086 10.1093/bioinformatics/bty560 PMC6129281

[36] Li H. Aligning sequence reads, clone sequences and assembly contigs with BWA-MEM. 2013. arXiv preprint arXiv:1303.3997.

[37] McKenna A, Hanna M, Banks E, Sivachenko A, Cibulskis K, Kernytsky A, et al. The genome analysis toolkit: a MapReduce framework for analyzing next-generation DNA sequencing data. Genome Res. 2010;20(9):1297–303. 20644199 10.1101/gr.107524.110 PMC2928508

[38] Wang K, Li M, Hakonarson H. ANNOVAR: functional annotation of genetic variants from high-throughput sequencing data. Nucleic Acids Res. 2010;38(16):e164-e. 20601685 10.1093/nar/gkq603 PMC2938201

[39] Danecek P, Auton A, Abecasis G, Albers CA, Banks E, DePristo MA, et al. The variant call format and VCFtools. Bioinformatics. 2011;27(15):2156–8. 21653522 10.1093/bioinformatics/btr330 PMC3137218

[40] Menegidio FB, Aciole Barbosa D, Goncalves RDS, Nishime MM, Jabes DL, de Costa Oliveira R, et al. Bioportainer Workbench: a versatile and user-friendly system that integrates implementation, management, and use of bioinformatics resources in Docker environments. GigaScience. 2019;8(4):giz041. 31222200 10.1093/gigascience/giz041 PMC6482343

[41] Hazbón MH, Brimacombe M, del Valle Bobadilla M, Cavatore M, Guerrero MI, Varma-Basil M, et al. Population genetics study of isoniazid resistance mutations and evolution of multidrug-resistant Mycobacterium tuberculosis. Antimicrob Agents Chemother. 2006;50(8):2640–9. 16870753 10.1128/AAC.00112-06 PMC1538650

[42] Sintchenko V, Chew WK, Jelfs PJ, Gilbert GL. Mutations in rpoB gene and rifabutin susceptibility of multidrug-resistant Mycobacterium tuberculosis strains isolated in Australia. Pathology. 1999;31(3):257–60. 10503273 10.1080/003130299105089

[43] Sechidis K, Tsoumakas G, Vlahavas I, editors. On the stratification of multi-label data. Joint European Conference on Machine Learning and Knowledge Discovery in Databases. Berlin, Heidelberg: Springer; 2011.

[44] Guo M-H, Liu Z-N, Mu T-J, Liang D, Martin RR, Hu S-M. Can Attention Enable MLPs To Catch Up With CNNs?. Comput Vis Med. 2021;7:283–8.

