# Correction: TB-DROP: deep learning-based drug resistance prediction of *Mycobacterium tuberculosis* utilizing whole genome mutations

**DOI:** 10.1186/s12864-026-13064-4

**Published:** 2026-07-01

**Authors:** Yu Wang, Zhonghua Jiang, Pengkuan Liang, Zhuochong Liu, Haoyang Cai, Qun Sun

**Affiliations:** 1 Key Laboratory of Bio-Resources and Eco-Environment of the Ministry of Education, College of Life Sciences, Sichuan University, Chengdu, 610064 China; 2 Zhejiang Yangshengtang Institute of Natural Medication Co., Ltd, Hangzhou, China; 3 Center of Growth, Metabolism and Aging, Key Laboratory of Bio Resources and Eco Environment of the Ministry of Education, College of Life Sciences, Sichuan University, Chengdu, 610064 China

**Correction: BMC Genomics25, 167 (2024)**

**https://doi.org/10.1186/s12864-024-10066-y**

Following publication of the original article it was reported that the figure captions were not paired with the correct figures.

The caption originally published with Fig. 1 is the caption of Fig. 5.

The caption originally published with Fig. 2 is the caption of Fig. 1.

The caption originally published with Fig. 3 is the caption of Fig. 2.

The caption originally published with Fig. 4 is the caption of Fig. 3.

The caption originally published with Fig. 5 is the caption of Fig. 4.

The correct figure captions are given below and the original article has been updated.

**Figure. 1** Summary of drug resistance status of all isolates.

**Figure. 2 **Loss curve. **A** Loss curve of training process; **B** Loss curve of validation process. Deepamr_inh represents the loss curve of the drug, isoniazid (INH), ethambutol (EMB), rifampicin (RIF), and pyrazinamide (PZA).

**Figure 3.** The workflow of TB-DROP.

**Figure 4.** TB-DROP user interface.

**Figure 5.** The representative architectures of four models, including the TB-DROP. The upper-left panel is the model architecture of WDNN, which comprises two parts: wide part and deep part. The model architecture implemented in TB-DROP (upper-right) is a deep neural network (DNN). The bottom-left panel is the model architecture of DeepAMR, which comprises encoder, decoder, and output layers. The model architecture of CNNGWP in the bottom-right panel is a classic convolutional neural network which consists of a convolutional layer and a pooling layer.

