# Supplementary material for: TB-DROP: deep learning-based drug resistance prediction of Mycobacterium tuberculosis utilizing whole genome mutations
Source: BMC Genomics. 2024 Feb 12;25:167. doi: 10.1186/s12864-024-10066-y (PMC10860279; doi:10.1186/s12864-024-10066-y)
Supplement: Supplementary file 1 — Additional file 1. Supplementary Materials. [file 12864_2024_10066_MOESM1_ESM.docx]

**TB-DROP: Deep Learning-based Drug Resistance Prediction of *Mycobacterium tuberculosis* utilizing Whole Genome Mutations**

**Supplementary Materials**

1. **Hyperparameter tuning process**

The hyperparameter tuning process for each of four models was presented below.

- 1. **WDNN**

Firstly, the original version of WDNN was tested using our whole genome mutations. Although the training loss curve declined steadily, the validation loss curve was U-shaped, which indicated overfitting existed. Therefore regularization techniques were applied. Next, the value of lambda of L2 regularizer was increased from 1e-8 to 1e-3. The overfitting was significantly improved inferred from the steady decline of training and validation loss curves. Thirdly, we attempted to increase the number of neurons of each layer. The number of neurons of all layers was 256 for the original version. It was enough for WDNN because the number of input features was 222. However, the number of our input features was very large and the small number of neurons might limit the learning capability of the model and couldn’t fit well. Therefore we increased the number of neurons of each layer. This led to the decreasing of learning speed and the performance of the model just changed slightly. Based on this fact, we gave up changing the number of neurons and turned to increasing the generalization ability of models from the aspect of regularization. Firstly, the L2 regularization was applied to the kernel and the bias in the dense layer at the same time. The prediction performance improved significantly. Next, the L1 regularization was applied to the kernel and the L2 regularization was applied to the bias. The reason we designed like this was because the L1 regularization could lead to most of weights equal to zero. This corresponded to the biological background that most mutations didn’t determine the drug-resistant phenotype. And L2 regularization could ensure that no bias would be too large which decreased the possibility of overfitting. At this time, the prediction effect of the model has been greatly improved. The result of this model was the final result of the WDNN based model. The final architecture of WDNN was presented in Figure 1 below.

- 1. **DeepAMR**

The original DeepAMR model required that each isolate had phenotypes for each drug. But our dataset couldn’t satisfy this requirement，lots of isolates missed phenotype for one or two drugs. Therefore, with the aid of WDNN’s custom loss function, the DeepAMR model was updated to be able to support the condition that not every isolate had phenotypes for each drug. The model could be trained with our dataset then but its overfitting was severe according to its U-shaped validation loss curve. Next, we focused on the overfitting problem and started from the part of the denoising autoencoder. Like the WDNN model, the original input features for DeepAMR were also very small. Hence the number of neurons in the denoising autoencoder was small. When we applied such a denoising autoencoder to our dataset, the number of input features was very large compared to the number of neurons. It required the denoising autoencoder learned to compress lots of features into a small set of feature. It was really difficult and would lose lots of information. Therefore, we increased the number of neurons in the denoising autoencoder and obtained significant improvement. The decoder’s output was almost the same as the input. Next, we started to consider how to improve overfitting occurred during predicting drug resistance of MTB based on the encoder’s output of the denoising autoencoder. The regularization method, parameter norm penalty, was applied firstly but failed. Many values of lambda were tested including very large ones (1e-3~1e-1) but no one could control the overfitting. And the metrics were always low (i.e. the sensitivity of each drug was around 0.7). In view of this, we gave up further tuning DeepAMR at this time. The final architecture of DeepAMR was presented in Figure 2 below.

- 1. **CNNGWP**

The first problem of applying CNNGWP to predict drug resistance of MTB was to modify it to enable it to predict qualitative traits, because its original design was to predict quantitative traits. The modification was to change the activation function of the output layer from linear to sigmoid and the loss function from mean squared error to binary cross entropy. Another update of CNNGWP was to incorporate the advantages of WDNN and DeepAMR: custom loss function and class-weight binary cross entropy and the MSSS cross validation strategy. Like WDNN and DeepAMR, the original CNNGWP underwent severe overfitting. Different from DeepAMR, the increasing of lambda value of the regularizer improved overfitting immediately and the predicting performance was also pretty good (i.e. the average sensitivity was around 0.85). Therefore, we further tuned this model from the aspects of L1 regularization lambda, Adam learning rate, number of convolution and pooling layers and number of filters. Finally, it achieved the second best performance. The final architecture of CNNGWP was presented in Figure 3 below.

- 1. **MLP**

Considering that 256 neurons were indeed too few, the number of neurons was increased to 1000 for each layer. The performance of the MLP model increased slightly. Then, we tested different value of dropout, different lambda value of the kernel and bias regularizer, and finally got the MLP model with the best performance. And this model was finally deployed in the first version of TBDRP. The final architecture of MLP was presented in Figure 4 below.
