# Supplementary material for: TB-DROP: deep learning-based drug resistance prediction of Mycobacterium tuberculosis utilizing whole genome mutations
Source: BMC Genomics. 2024 Feb 12;25:167. doi: 10.1186/s12864-024-10066-y (PMC10860279; doi:10.1186/s12864-024-10066-y)
Supplement: Supplementary file 2 — Additional file 2. Separate loss curves for each model. X-axis represents epochs and Y-axis represents loss values. Because each model converges after different epochs, the ranges of X-axis of each model are different from each other. This file corresponds to Fig. 3 in the manuscript. The pictures below are used to show more details of training process so they are presented separately. [file 12864_2024_10066_MOESM2_ESM.docx]

**Additional file 2**

Separate loss curves for each model. X-axis represents epochs and Y-axis represents loss values. Because each model converges after different epochs, the ranges of X-axis of each model are different from each other. This file corresponds to Figure 3 in the manuscript. The pictures below are used to show more details of training process so they are presented separately.


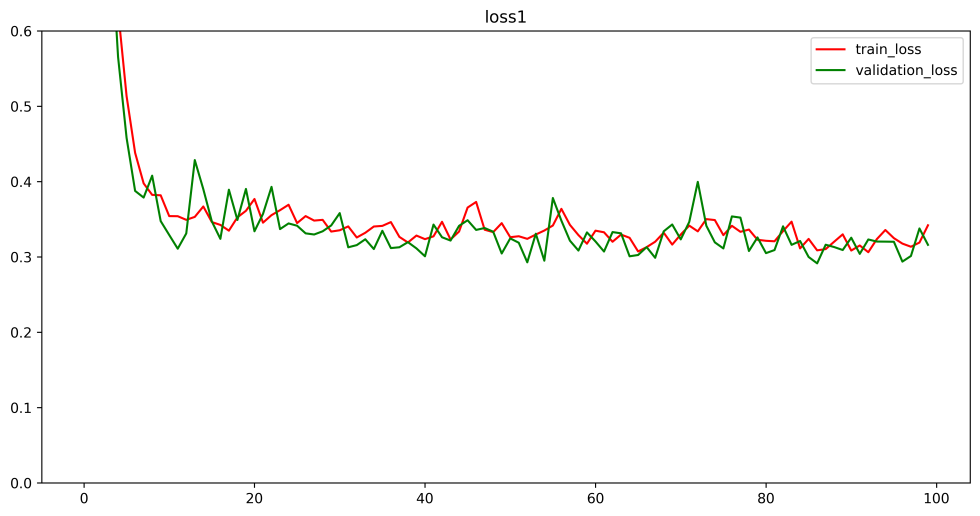


a. wdnn_modified loss curve


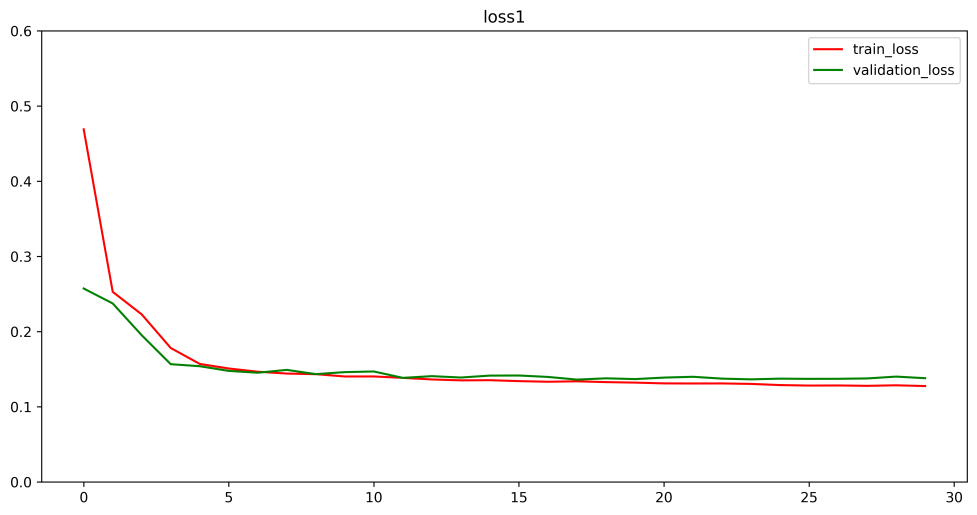


b. cnngwp_modified loss curve


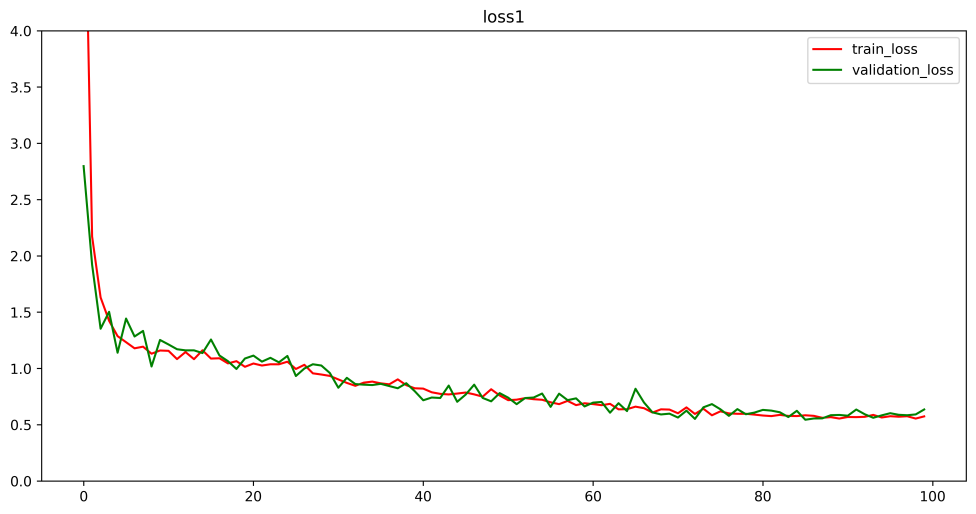


c. MLP loss curve


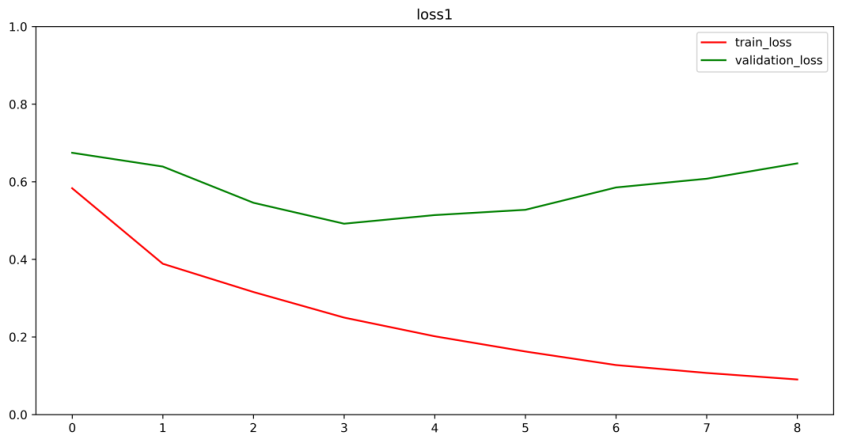


d. deepamr_modified_1(ISONIAZID)


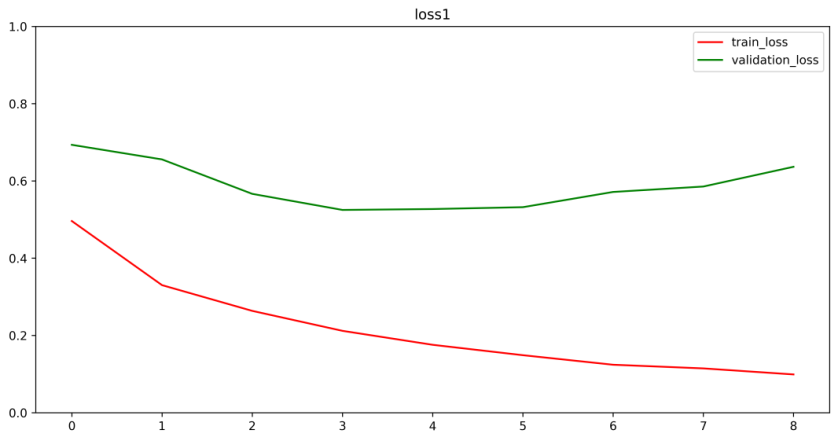


e. deepamr_modified_2(RIFAMPICIN)


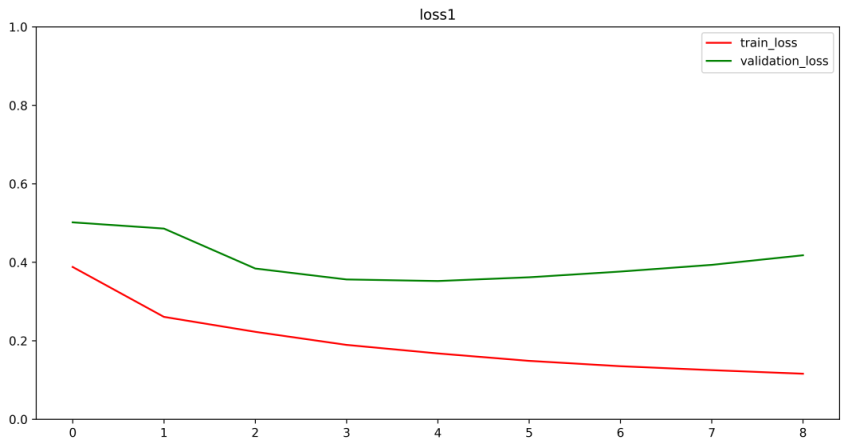


f. deepamr_modified_3(RIFAMPICIN)


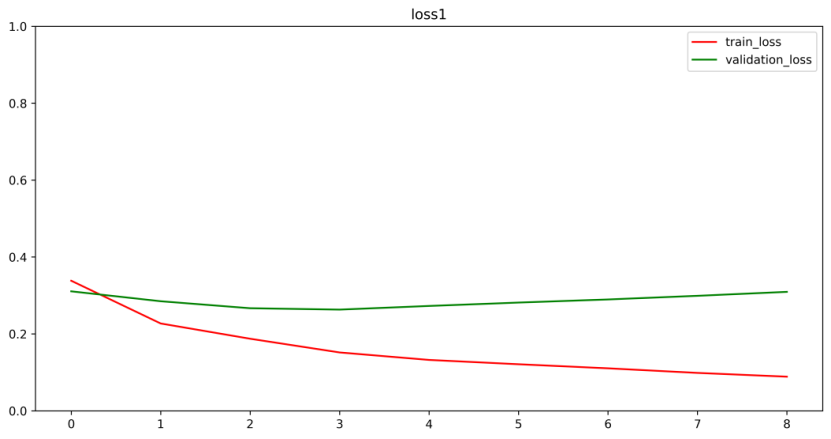


g. deepamr_modified_4(RIFAMPICIN)
